# DDB1 maintains intestinal homeostasis by preventing cell cycle arrest

**DOI:** 10.1186/s13619-022-00119-6

**Published:** 2022-06-01

**Authors:** Lianzheng Zhao, Hongwei Liao, Xiaodan Wang, Ye-Guang Chen

**Affiliations:** 1 The State Key Laboratory of Membrane Biology, Tsinghua-Peking Center for Life Sciences, School of Life Sciences, Tsinghua University, Beijing, 100084 China; 2 Guangzhou Laboratory, Guangzhou, China

**Keywords:** DDB1, Intestine, Homeostasis, Cell cycle

Dear Editor,

The intestinal epithelium is a rapidly self-renewing tissue for absorbing nutrients and providing barrier functions, and its homeostasis is orchestrated by several signaling pathways (Vermeulen and Snippert, 2014; Zhu et al., 2021). Growing evidence demonstrates the importance of cell cycle regulation in intestinal homeostatic maintenance (McKernan and Egan, 2015). Here, we report that the E3 ubiquitin ligase adaptor DDB1 (Damaged DNA Binding Protein 1) is highly expressed in the intestinal epithelium and regulates the intestinal homeostasis by preventing cell cycle arrest.

DDB1 was highly expressed throughout the intestinal epithelium, and also distributed in the lamina propria and muscularis propria (Fig. 1A and S1A). To assess DDB1 function in the intestinal epithelium, we crossed *DDB1*^*fl/fl*^ mice with *Villin-Cre* mice to ablate DDB1 in the intestinal epithelium. The homozygous mice were not obtained, indicating that DDB1 plays an essential role in embryonic *Villin*^+^ cells. Next we generated the inducible *DDB1* knockout (KO) *DDB1*^*fl/fl*^*;Villin-CreERT2* mice, and five-day tamoxifen (TAM) administration led to the complete ablation of DDB1 throughout the intestinal epithelium (Fig. 1A, S1A and S1B). The KO mice exhibited rapid weight loss and died before day 9 (the day with first TAM injection was regarded as day 0, Fig. S1C and S1D), indicating that DDB1 is vital for the maintenance of intestinal homeostasis. Compared to the *DDB1*^*fl/fl*^ group (Ctrl), the small intestine of KO mice showed hemorrhage with shortened length, while the length of large intestine was unaltered (Fig. 1B and S1E). Histologically, the loss of DDB1 led to the collapse of small intestinal epithelium with deteriorating crypts (Fig. 1C and S2A), whereas the large intestinal structures exhibited moderate changes (Fig. S2B and S2C), suggesting that DDB1 plays different roles in different intestinal segments. Before the tissue collapse at day 4, the Ki67^+^ proliferating cells in the transient amplifying region of crypts were already decreased in the small intestine (Fig. 1D, S3A and S3B), while this change was delayed in the large intestine at day 6 (Fig. S3C and S3D). Moreover, the TUNEL assay revealed that cell death was increased in the KO small intestine (Fig. S3E and S3F). Therefore, the decreased cell proliferation and increased death would contribute to the disruption of homeostasis.

**Fig. 1:**
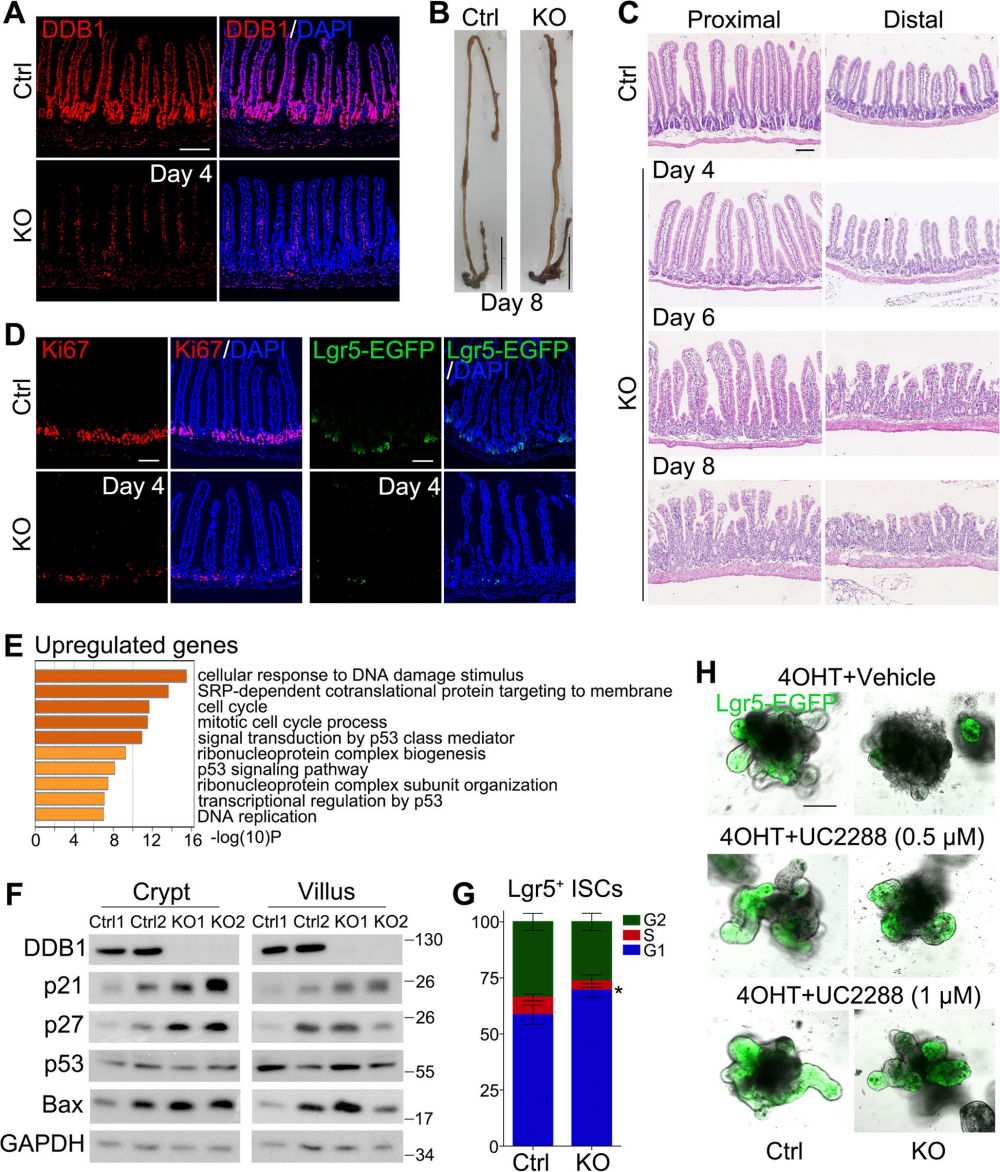
DDB1 maintains intestinal homeostasis by preventing cell cycle arrest. **A** Immunofluorescence (IF) staining to detect DDB1 expression in Ctrl (*DDB1*^*fl/fl*^) and KO (*DDB1*^*fl/fl*^*;Villin-CreERT2*) small intestine at day 4 after the first TAM injection. The day with first TAM injection is regarded as day 0. Scale bar, 100 μm. **B** Representative images of Ctrl and KO intestine at day 8. Scale bar, 5 cm. The length of intestine is quantified in Fig. S1E. **C** Hematoxylin and eosin (H&E) staining of proximal and distal small intestine from Ctrl and KO mice at day 4, day 6 and day 8. Scale bar, 100 μm. The viable crypt number is quantified in Fig. S2A. **D** IF staining of Ki67 and endogenous Lgr5-EGFP in Ctrl and KO small intestine at day 4. Scale bar, 100 μm. The number of Ki67^+^ cells and Lgr5^+^ crypts are shown in Fig. S3B and S4A respectively. **E** Gene ontology (GO) analysis of upregulated genes in KO small intestinal crypts at day 2, obtained from RNA-seq (*n* = 2). **F** Immunoblotting of small intestinal crypts and villi from Ctrl and KO mice at day 2. **G** Cell cycle analysis of Lgr5^+^ ISCs from Ctrl and KO mice at day 2. Data are presented as mean ± SD (*n* = 3). Student’s t-test, *P < 0.05. **H** UC2288 treatment of Lgr5-EGFP labeled small intestinal organoids for 4 days with or without *DDB1* deletion. *DDB1* deletion in organoids is induced by 4-OHT. Scale bar, 100 μm. The budding number and Lgr5^+^ cell ratio of organoids are shown in Fig. S6B and S6C, respectively

Next we examined whether *DDB1* deficiency would affect stemness. The *DDB1*^*fl/fl*^*;Villin-CreERT2;Lgr5-EGFP-IRES-CreERT2* (*Lgr5-EGFP*) mice were used to label intestinal stem cells (ISCs) and treated with TAM as above. At day 4, the number of Lgr5^+^ crypts in the KO small intestine was reduced dramatically (Fig. 1D and S4A), and the expressions of other ISC markers including *Olfm4* and *Ascl2* were also downregulated (Fig. S4B). Consistently, the decrease of Lgr5^+^ ISCs was also observed in cultured organoids after 4-hydroxytamoxifen (4-OHT) induced knockout in vitro (Fig. S4C and S4D). We also examined differentiated cells after *DDB1* deletion. The immunofluorescence staining unveiled the reduced number of Chga^+^ enteroendocrine cells and Muc2^+^ goblet cells at day 6, while the Lyz^+^ Paneth cells were unchanged (Fig. S5).

To explore the molecular mechanism underlying the role of DDB1 in the intestinal homeostasis, the small intestinal crypts of Ctrl and KO mice at the early stage (day 2) were isolated and subjected to RNA-sequencing. The upregulated genes induced by *DDB1* deficiency were enriched in the cell cycle process and p53 signaling (Fig. 1E and Table S1), reminiscent of the canonical function of DDB1 in cell cycle in other tissues (Cang et al., 2007; Zhao et al., 2020). Indeed, the immunoblotting verified the increased expression of p21 and p27 in KO small intestine (Fig. 1F), both of which are cyclin-dependent kinase (CDK) inhibitors to induce cell cycle arrest and targeted for degradation by DDB1 as an adaptor for Cul4A E3 ubiquitin ligase (Abbas et al., 2008; Bondar et al., 2006). Consistently, the cell cycle analysis of Lgr5^+^ ISCs showed that DDB1 KO led to more ISCs arrested at the G1 phase (Fig. 1G). In addition, the pro-apoptotic factor Bax, downstream of p53 signaling, was also upregulated (Fig. 1F), consistent with the increased cell death. Loss of DDB1 in organoids also induced the upregulation of *p21* mRNA (Fig. S6A), indicating that p21 is an important mediator of DDB1 action. Indeed, the p21 inhibitor UC2288 could partially rescue the DDB1 KO-induced death and increase budding number and ISC number in the organoids (Fig. 1H, S6B and S6C). Intestinal epithelium hyperplasia was observed in *ALK3*^*fl/fl*^*;Villin-CreERT2* mice, as indicated by the elongated crypts and increased proliferation zone (Qi et al., 2017) (Fig. S7A). *DDB1* knockout still inhibited the proliferation in the double knockout mice (*DDB1*^*fl/fl*^*;ALK3*^*fl/fl*^*;Villin-CreERT2*) (Fig. S7B and S7C), confirming that DDB1 plays a critical role in regulation of cell proliferation.

In summary, using the genetic mouse models and organoids, we demonstrate that DDB1 plays a critical role in the fast homeostatic renewal of the intestinal epithelium, which is achieved by reducing CDK inhibitor expression, preventing cell cycle arrest in the G1 phase and thus ensuring normal cell proliferation.

## Supplementary Information

**Additional file 1: Supplementary Methods. Figure S1.** *DDB1* deficiency leads to mouse lethality with shortened small intestine. **Figure S2.** *DDB1* deletion causes reduced crypts in the small intestine. **Figure S3.** *DDB1* deficiency impairs cell proliferation and enhances cell death in the intestine. **Figure S4.** Ablation of DDB1 reduces Lgr5^+^ ISCs. **Figure S5.** Decrease of goblet cells and enteroendocrine cells in the small intestine upon *DDB1* deletion. **Figure S6.** Inhibition of p21 by UC2288 partially rescues the phenotypes caused by *DDB1* deletion in organoids. **Figure S7.** *DDB1* deletion inhibits cell proliferation induced by ALK3 KO. **Table S1.** Differentially expressed genes of small intestinal crypts after *DDB1* deletion at day 2.

## Data Availability

Data and material are available from the corresponding author on reasonable request.

## Abbreviations

4-OHT: 4-hydroxytamoxifen
Alk3: Bone morphogenetic protein receptor, type 1A
Ascl2: Achaete-scute family bHLH transcription factor 2
Bax: BCL2-associated X protein
CDK: Cyclin-dependent kinase
Chga: Chromogranin A
Ctrl: Control
Cul4A: Cullin 4A
DDB1: Damaged DNA binding protein 1
ISC: Intestinal stem cell
KO: Knockout
Lgr5: Leucine rich repeat containing G protein coupled receptor 5
Lyz: Lysozyme
Muc2: Mucin 2
Olfm4: Olfactomedin 4
p21: Cyclin-dependent kinase inhibitor 1A
p27: Cyclin-dependent kinase inhibitor 1B
TAM: Tamoxifen
TUNEL: TdT-mediated dUTP Nick-End Labeling
