# Supplementary material for: DDB1 maintains intestinal homeostasis by preventing cell cycle arrest
Source: Cell Regen. 2022 Jun 1;11:18. doi: 10.1186/s13619-022-00119-6 (PMC9156607; doi:10.1186/s13619-022-00119-6)
Supplement: Supplementary file 1 — Additional file 1: Supplementary Methods. Figure S1. DDB1 deficiency leads to mouse lethality with shortened small intestine. Figure S2. DDB1 deletion causes reduced crypts in the small intestine. Figure S3. DDB1 deficiency impairs cell proliferation and enhances cell death in the intestine. Figure S4. Ablation of DDB1 reduces Lgr5+ ISCs. Figure S5. Decrease of goblet cells and enteroendocrine cells in the small intestine upon DDB1 deletion. Figure S6. Inhibition of p21 by UC2288 partially rescues the phenotypes caused by DDB1 deletion in organoids. Figure S7. DDB1 deletion inhibits cell proliferation induced by ALK3 KO. Table S1. Differentially expressed genes of small intestinal crypts after DDB1 deletion at day 2. [file 13619_2022_119_MOESM1_ESM.zip › M-DDB1-SI-Cell Regeneration-finalR1.pdf]

Supplementary information

DDB1 maintains intestinal homeostasis by preventing cell cycle arrest

Lianzheng Zhao[1], Hongwei Liao[1], Xiaodan Wang[1], Ye-Guang Chen[1,2,*]

Supplementary Methods

Supplementary Figures: S1-S7

Supplementary Table: S1

Supplementary Methods

Mice

Villin-Cre mice and *Lgr5-EGFP-IRES-CreERT2* (*Lgr5-EGFP*) mice were obtained from Jackson Laboratory. *Villin-CreERT2* mice (el Marjou et al., 2004), $ALK3^{fl/fl}$ mice (Mishina et al., 2002) and $DDB1^{fl/fl}$ mice (Cang et al., 2006) were kindly provided by Drs. Sylvie Robine, Yuji Mishina and Yong Cang, respectively. Littermates were used in all experiments. For Cre induction, mice were intraperitoneally injected with tamoxifen dissolved in sunflower oil at 20 mg/ml for 5 consecutive days. All mice studies were performed in accordance with the relevant guidelines and under the approval of the Institutional Animal Care and Use Committee of Tsinghua University.

Histological analyses

For frozen sections, the intestine was isolated from indicated mice, washed with cold PBS and fixed in 4% formaldehyde for 1 h at room temperature. Then the tissues were soaked into 20% sucrose overnight at 4 ^{o}C followed by OCT (Optimal Cutting Temperature) compound (Sakura) embedding, frozen at -80 ^{o}C and sectioned. For paraffin sections, the isolated intestine were fixed in 4% formaldehyde overnight at 4 ^{o}C, then paraffin embedded and sectioned. For immunofluorescence, frozen sections were soaked into PBS to remove OCT, followed by antigen retrieval with citrate buffer in a microwave oven for 15 min. Then the sections were permeabilized with 0.1% Triton X-100 at 4 ^{o}C for 15 min, and blocked with 3% BSA + 0.01% Triton X-100 at room temperature for 1 h. The sections were incubated with the primary

antibody at 4 ^{o}C overnight. The fluorescein-labelled secondary antibodies (Life Technologies, 1:300) with DAPI were applied for 1 h at room temperature. Images were obtained with an Olympus FV3000 Laser Scanning Microscope. The following antibodies were used: rabbit anti-DDB1 (Abcam, ab109027, 1:100), rabbit anti-Ki67 (Abcam, ab15580, 1:300), rabbit anti-Muc2 (Abcam, ab272692, 1:200), rabbit anti-Lyz (Abcam, ab108508, 1:200) and goat anti-Chga (Santa Cruz, sc-1488, 1:300). For H&E staining, paraffin sections were de-paraffinized in xylene and graded alcohols, then stained with hematoxylin and eosin according to the manufacturer's instructions (C0205S, Beyotime). Images were obtained with a slide scanning system (KF-PRO-120, Jiang Feng Technology).

TUNEL assay

Paraffin sections were de-paraffinized in xylene and graded alcohols, then subjected to cell death assessment using the TUNEL cell death detection kit (Roche).

Intestinal crypt isolation and organoid culture

Intestinal crypts were isolated and cultured as previously described (Qi et al., 2017).

Cell cycle and cell ratio analyses

For cell cycle analysis, crypts isolated from indicated mice were dissociated in TrypLE (Invitrogen) for 30 min at 37 ^{o}C. The single cell suspension was washed twice with pre-cooling PBS and then fixed in pre-cooling 70% ethyl alcohol overnight

at 4 ^{o}C. After washing with pre-cooling PBS twice, the cells were resuspended gently using propidium staining solution (Beyotime) and then incubated at 37 ^{o}C for 30 min. The contents of stained DNA of EGFP^{+} (Lgr5^{+}) ISCs were examined by flow cytometry (CytoFlex LX, Beckman Coulter) and analyzed by Kaluza Analysis software. For cell ratio analysis, organoids were dissociated in TrypLE (Invitrogen) for 20 min at 37 ^{o}C. The single cell suspension was passed through 40 μm cell strainer (BD Biosciences) and centrifuged for 3 min at 500g. The cells were resuspended gently using propidium staining solution (Beyotime) and tested by flow cytometry (CytoFlex LX, Beckman Coulter) to indicate the ratio of EGFP^{+} (Lgr5^{+}) ISCs.

RNA extraction and qRT-PCR

Total RNA from crypts or organoids was extracted using TRIzol (Invitrogen). cDNA was prepared using Revertra Ace (Toyobo). qRT-PCR was performed in triplicates on a LightCycler 480 (Roche) with *Gapdh* as the reference gene. Data were analyzed according to the ΔCT method. The following primers were used: *Lgr5*, 5'-CGGGACCTTGAAGATTTCCT-3' and 5'-GATTCGGATCAGCCAGCTAC-3'; *Ascl2*, 5'-GCCTGACCAAATGCCAAGTG-3' and 5'-ATTTCCAAGTCCTGATGCTGC-3'; *Olfm4*, 5'-CGAGACTATCGGATTCGCTATG-3' and 5'-TTGTAGGCAGCCAGAGGGAG-3'; *p21*, 5'-AAGAGGCCCAGTACTTCC-3' and 5'-TCTTGCAGAAGACCAATCTG-3'; *p27*, 5'-TAATTGGGTCTCAGGCAAAC-3' and 5'-GGGAACCGTCTGAAACATT-3';

p53, 5’-TATCCGGGTGGAAGGAAA-3’ and 5’-CCAGTGTGATGATGGTAAGG-3’; *Ddb1*, 5’-TGGCCAATAACAGCACTCTC-3’ and 5’-TAGTGCCTCCACTGCTATCT -3’; *Gapdh*, 5’-AAGAAGGTGGTGAAGCAG-3’ and 5’-TCATACCAGGAAATGAGC-3’.

Bulk RNA-seq

Total RNA from crypts (day 2) was extracted using RNeasy Mini Kit (Qiagen) and converted into cDNA libraries using the Ovation RNA-Seq System V2 kit (NuGEN). High-throughput sequencing was performed using the Illumina HiSeq 2000. The RNA-seq was carried out with two biological replicates. Genes with fold changes of 1.5 were regarded as differentially expressed genes. GO analysis was performed with Metascape (https://metascape.org/).

Immunoblotting

Isolated crypts and scraped villi were lysed in TNE buffer with protease inhibitors (Roche). Then total protein was subjected to SDS-PAGE and then transferred to nitrocellulose membranes (Bio Trace). The membranes were blocked in 5% skim milk and then incubated with the primary antibody overnight at 4 ^{o}C, followed by incubation with a secondary antibody conjugated with horse-radish peroxidase (HRP) for 1 h at room temperature. The following antibodies were used: rabbit anti-DDB1 (Abcam, ab109027, 1:10,000); mouse anti-p21 (Cell Signaling Technology, 2946s,

1:1,000); rabbit anti-p27 (Proteintech, 25614-1-AP, 1:1,000); mouse anti-p53 (Santa Cruz, sc-98, 1:1,000); rabbit anti-Bax (Proteintech, 50599-2-lg, 1:1,000); mouse anti-GAPDH (Abcam, ab8245, 1:3,000) and HRP-conjugated mouse IgG or rabbit IgG (Cell Signaling Technology, 7074s, 7076s, 1:10,000).

Statistical analysis

All experiments were carried out with at least three biological replicates unless noted specially. Data are presented as mean ± SD. Statistical differences were determined by Student's t-test. Data from qRT-PCR were analyzed using a two-way analysis of variance (ANOVA). Significance was accepted at P-values less than 0.05.

References

Cang, Y., Zhang, J., Nicholas, S.A., Bastien, J., Li, B., Zhou, P., and Goff, S.P. (2006). Deletion of DDB1 in mouse brain and lens leads to p53-dependent elimination of proliferating cells. Cell *127*, 929-940.

el Marjou, F., Janssen, K.P., Chang, B.H., Li, M., Hindie, V., Chan, L., Louvard, D., Chambon, P., Metzger, D., and Robine, S. (2004). Tissue-specific and inducible Cre-mediated recombination in the gut epithelium. Genesis *39*, 186-193.

Mishina, Y., Hanks, M.C., Miura, S., Tallquist, M.D., and Behringer, R.R. (2002). Generation of Bmpr/Alk3 conditional knockout mice. Genesis *32*, 69-72.

Qi, Z., Li, Y., Zhao, B., Xu, C., Liu, Y., Li, H., Zhang, B., Wang, X., Yang, X., Xie, W.*, et al.* (2017). BMP restricts stemness of intestinal Lgr5+ stem cells by directly suppressing their signature genes. Nature Communications *8*.

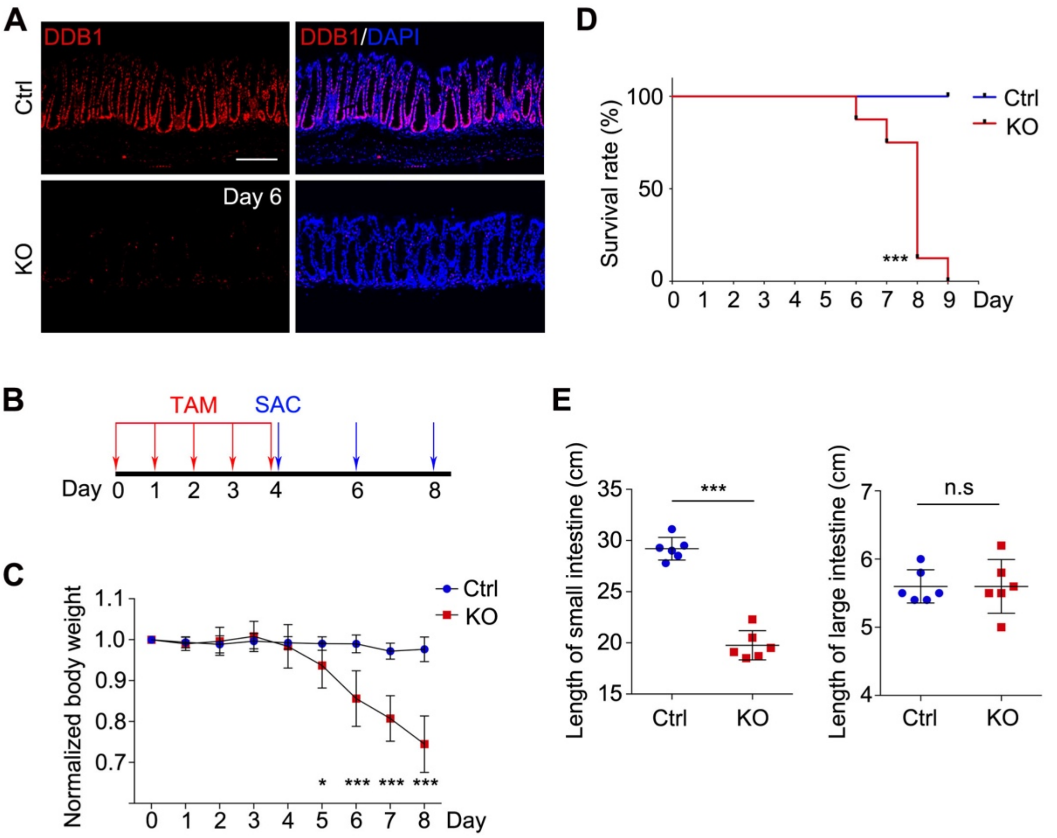

Fig. S1 *DDB1* deficiency leads to mouse lethality with shortened small intestine. **A** IF staining to detect DDB1 expression in Ctrl and KO large intestine at day 6. Scale bar, 100 μm. **B** Experimental scheme for *DDB1* deletion in the small intestinal epithelium. TAM: tamoxifen; SAC: sacrifice. **C** Body weight changes after *DDB1* deletion. The body weight of KO mice is normalized to Ctrl mice. Data are presented as mean ± SD (n = 6). Student's t-test, **P* < 0.05, ****P* < 0.001. **D** Survival rate of Ctrl and KO mice (n = 8). Log rank test, ****P* < 0.001. **E** Length of small and large intestine from Ctrl and KO mice at day 8, related to Fig. 1B. Data are presented as mean ± SD (n = 6). Student's t-test, ****P* < 0.001, n.s, not significant.

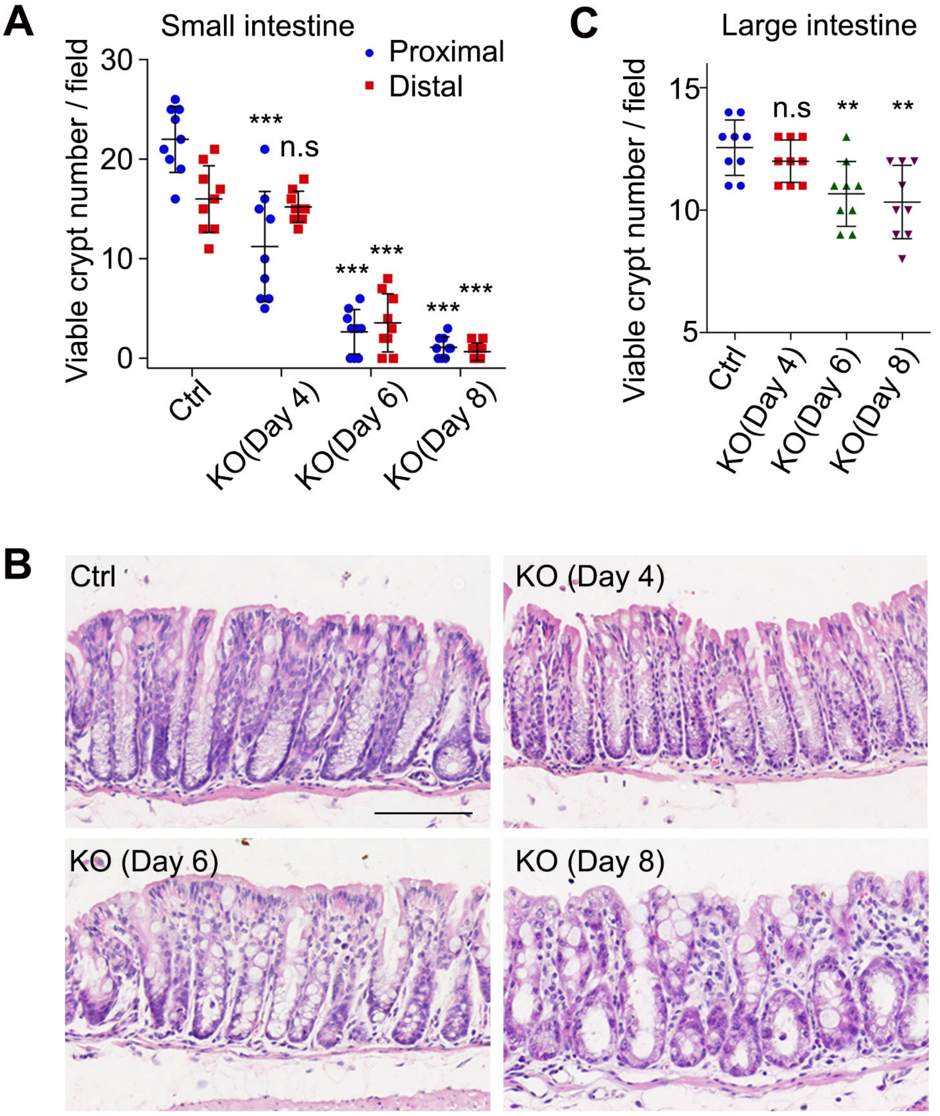

Fig. S2 *DDB1* deletion causes reduced crypts in the small intestine. **A** Viable crypt number of Ctrl and KO small intestine at day 4, day 6 and day 8, related to Fig. 1C. **B** H&E staining of large intestine from Ctrl and KO mice at day 4, day 6 and day 8. Scale bar, 100 μm. **C** Viable crypt number of Ctrl and KO large intestine at day 4, day 6 and day 8. Data are presented as mean ± SD (n = 3). Student's t-test, $^{**}P < 0.01$, $^{***}P < 0.001$, n.s, not significant.

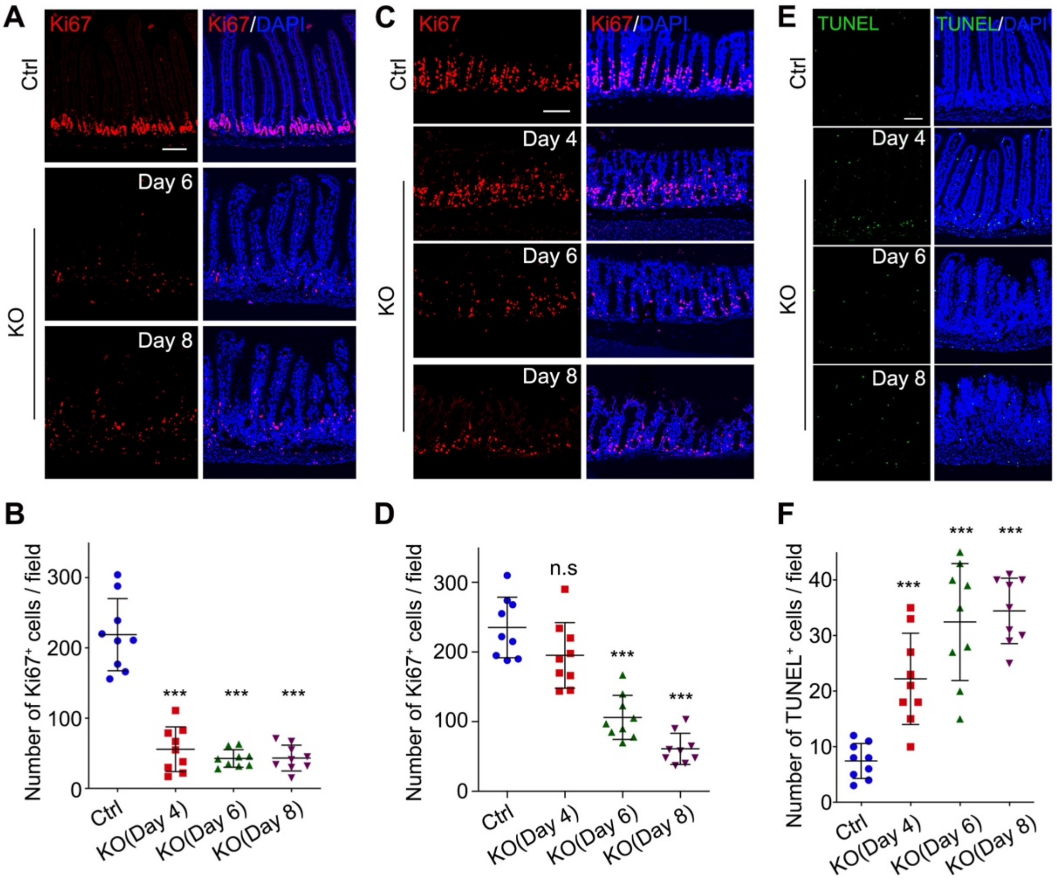

Fig. S3 *DDB1* deficiency impairs cell proliferation and enhances cell death in the intestine. **A** IF staining of Ki67 in Ctrl and KO small intestine at day 6 and day 8. Scale bar, 100 μm. **B** Number of Ki67$^+$ cells in Ctrl and KO small intestine at day 4, day 6 and day 8, related to Fig. 1D. **C** IF staining of Ki67 in Ctrl and KO large intestine at day 4, day 6 and day 8. Scale bar, 100 μm. **D** Number of Ki67$^+$ cells in Ctrl and KO large intestine at day 4, day 6 and day 8. **E** TUNEL staining of Ctrl and KO small intestine at day 4, day 6 and day 8. Scale bar, 100 μm. **F** Number of TUNEL$^+$ cells in Ctrl and KO small intestine at day 4, day 6 and day 8. Data are presented as mean ± SD (n = 3). Student's t-test, ***$P < 0.001$, n.s, not significant.

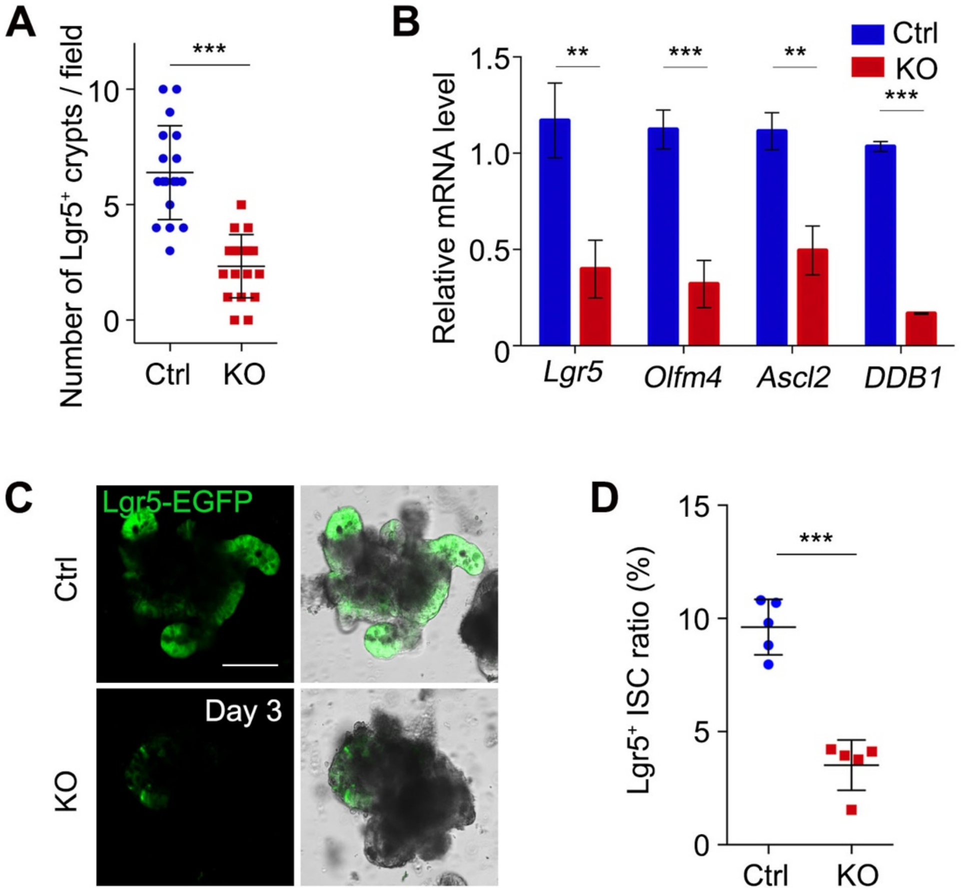

Fig. S4 Ablation of DDB1 reduces $Lgr5^+$ ISCs. **A** Number of $Lgr5^+$ crypts in Ctrl and KO small intestine at day 4 (n = 3), related to Fig. 1D. **B** Small intestinal crypts of Ctrl and KO mice at day 4 (n = 3) were isolated for qRT-PCR analysis of stem cell marker genes. **C** Lgr5-EGFP labeled small intestinal organoids derived from Ctrl and KO mice were treated by 4-OHT for 3 days to induce *DDB1* deletion *in vitro*. Scale bar, 100 μm. **D** Ratio of $Lgr5^+$ ISCs from small intestinal organoids after *DDB1* deletion for 3 days (n = 5), analyzed by flow cytometry. Data are presented as mean ± SD. Student's t-test (A and D), two-way ANOVA test (B), ***P* < 0.01, ****P* < 0.001.

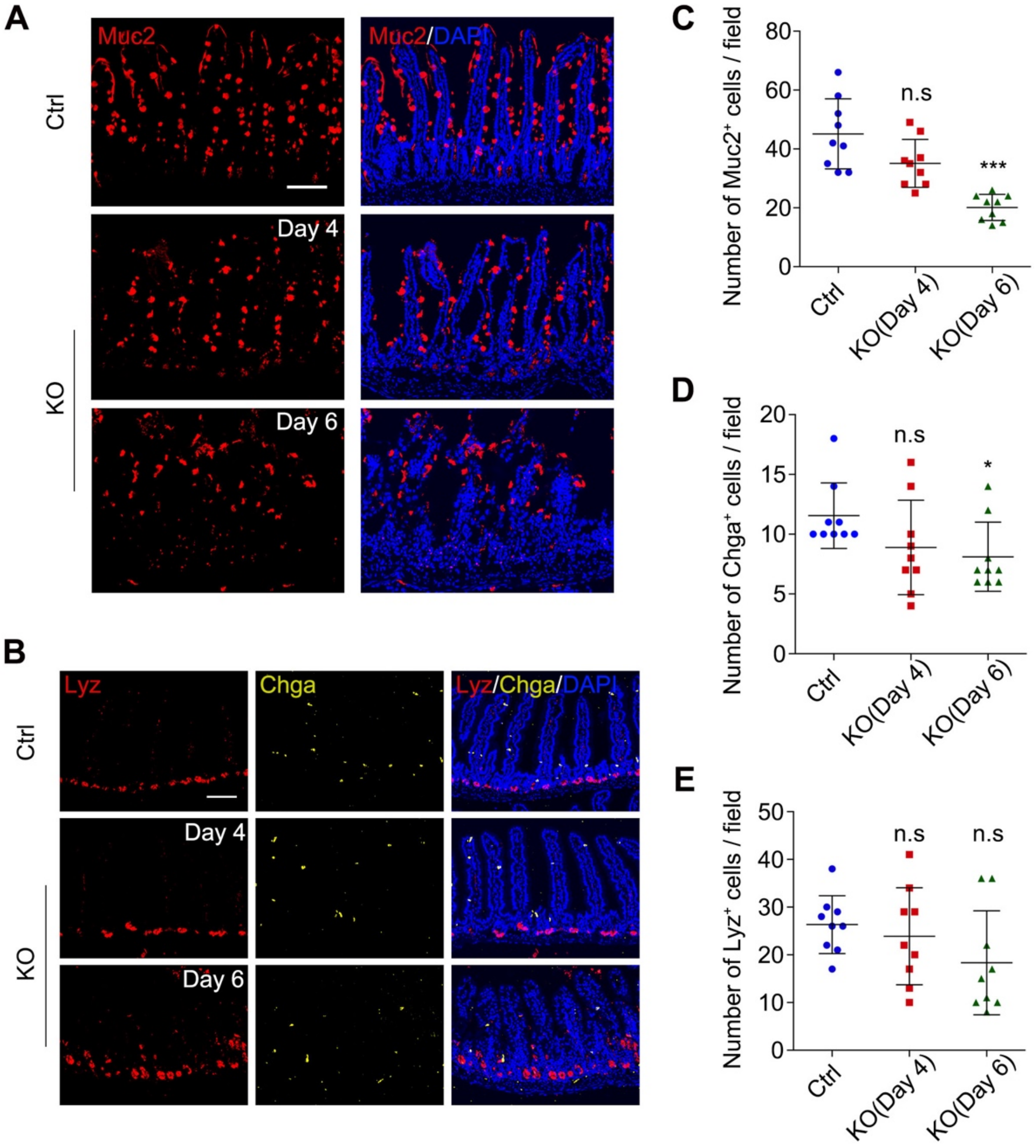

Fig. S5 Decrease of goblet cells and enteroendocrine cells in the small intestine upon *DDB1* deletion. **A** and **B** IF staining of Muc2 (A), Chga and Lyz (B) in Ctrl and KO small intestine at day 4 and day 6. Scale bar, 100 µm. **C**-**E** Number of $Muc2^+$ goblet cells (C), $Chga^+$ enteroendocrine cells (D) and Lyz^+ Paneth cells (E) in Ctrl and KO small intestine at day 4 and day 6. Data are presented as mean ± SD (n = 3). Student's t-test, **P* < 0.05, ****P* < 0.001, n.s, not significant.

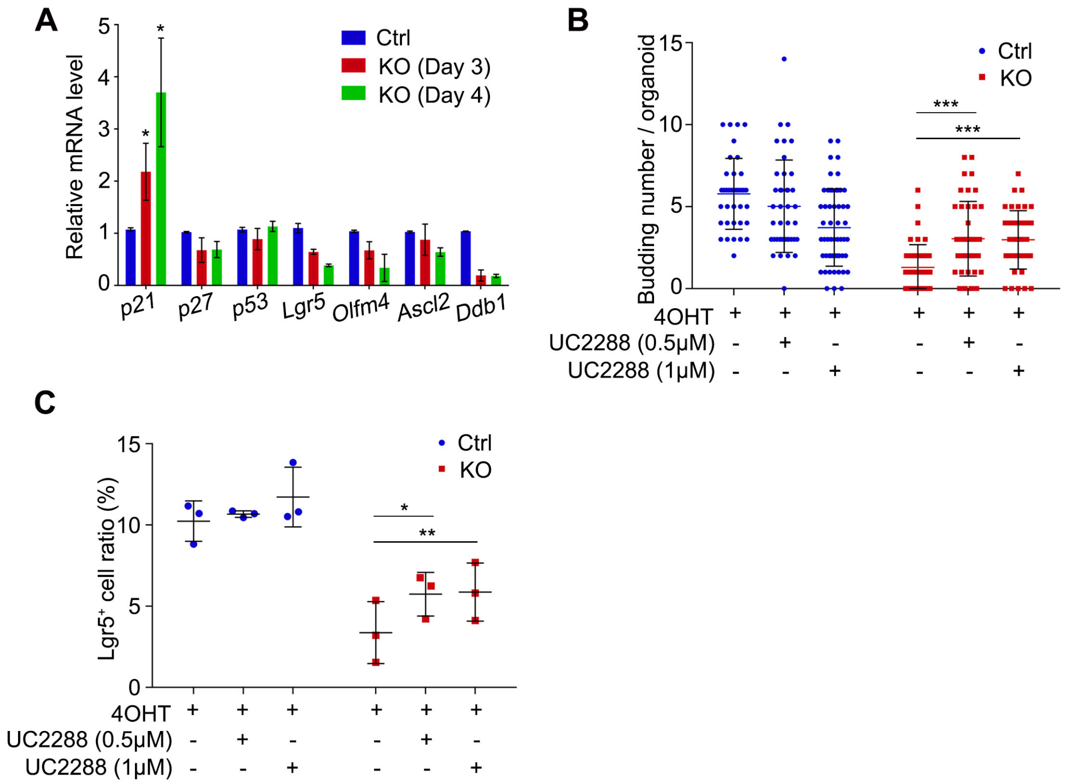

Fig. S6 Inhibition of p21 by UC2288 partially rescues the phenotypes caused by *DDB1* deletion in organoids. **A** qRT-PCR analysis of Ctrl and KO small intestinal organoids cultured for 3 or 4 days. **B** Budding number of Ctrl and KO small intestinal organoids treated with UC2288 for 4 days. **C** Lgr5^{+} ISC ratio of Ctrl and KO small intestinal organoids treated with UC2288 for 4 days, analyzed by flow cytometry. *DDB1* deletion in organoids was induced by 4-OHT. Data are presented as mean ± SD. Two-way ANOVA test (A), Student's t-test (B and C), $*P < 0.05$, $**P < 0.01$, $***P < 0.001$.

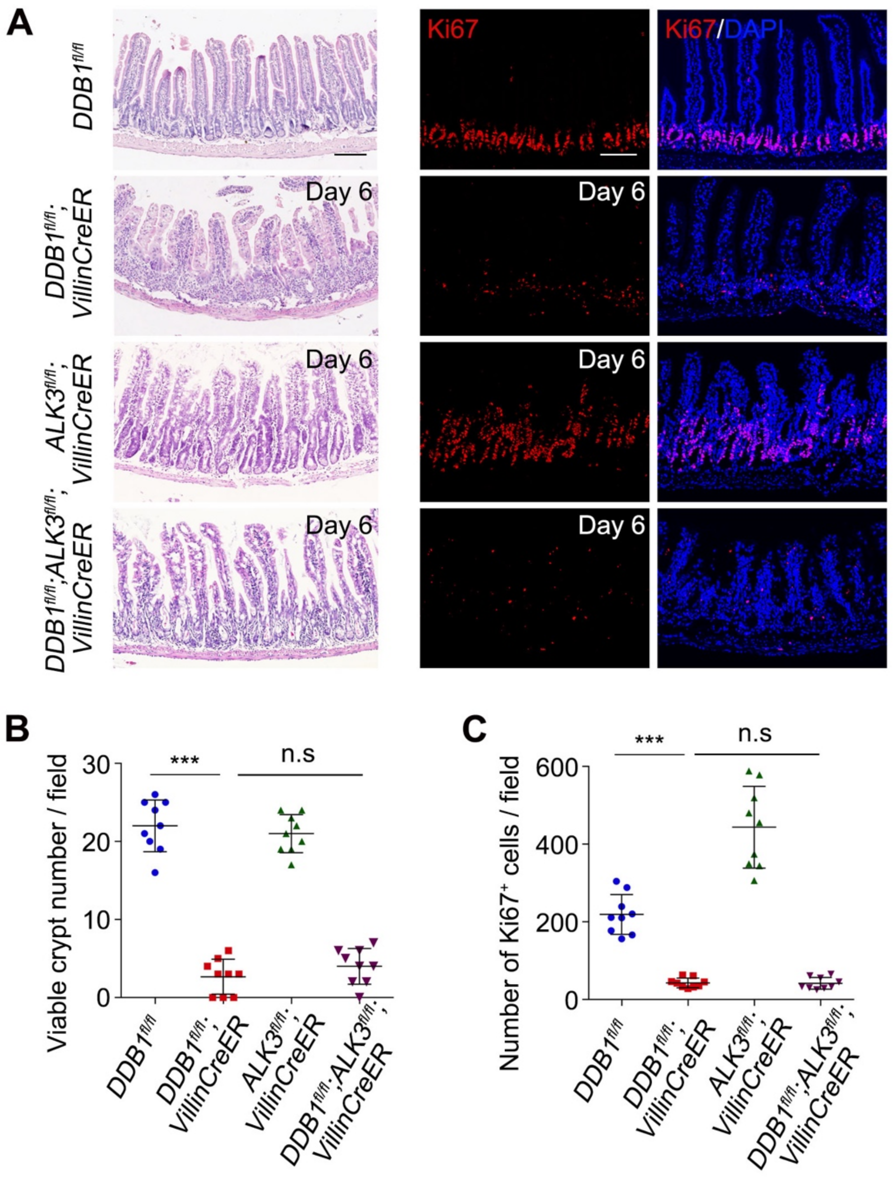

Fig. S7 *DDB1* deletion inhibits cell proliferation induced by ALK3 KO. **A** H&E staining and Ki67 staining of small intestine from indicated mice at day 6 after the first TAM injection. The day with first TAM injection is regarded as day 0. Scale bar, 100 μm. **B** Viable crypt number of small intestine from indicated mice at day 6. **C** Number of Ki67^{+} cells of small intestine from indicated mice at day 6. Data are presented as mean ± SD (n = 3). Student's t-test, ***$P < 0.001$, n.s, not significant.

Table S1 Differentially expressed genes of small intestinal crypts after *DDB1* deletion at day 2.
